# A rare case of sweat gland carcinoma of the scalp with neuroendocrine differentiation: Primary cutaneous malignancy versus breast cancer metastasis

**DOI:** 10.1016/j.jdcr.2025.07.014

**Published:** 2025-08-08

**Authors:** Olivia R. Negris, Jawad A. Aqeel, May P. Chan, Kelly L. Harms, Elisabeth A. Pedersen

**Affiliations:** a Department of Internal Medicine, University of Chicago – Northshore, Chicago, Illinois; b Department of Dermatology, University of Michigan Medicine, Ann Arbor, Michigan; c Department of Pathology, University of Michigan Medicine, Ann Arbor, Michigan

**Keywords:** breast cancer, low-grade neuroendocrine carcinoma of the skin, metastasis, sweat gland carcinoma with neuroendocrine differentiation

## Introduction

Sweat gland carcinoma with neuroendocrine differentiation (SCAND) is a rare neuroendocrine tumor of the skin. This disease concept was previously reported as a variant of low-grade neuroendocrine carcinoma of the skin.^1^, ^2^, ^3^ In 2022, the terminology of SCAND was proposed for cutaneous tumors with apocrine/eccrine differentiation and relatively aggressive biological behavior.^4^

SCAND most commonly occurs in older males along the milk lines of the trunk and is composed of nested tumor cells in the dermis with neuroendocrine differentiation and estrogen receptor (ER) positivity and may mimic breast cancer metastases.^4^, ^5^, ^6^ It can metastasize to lymph nodes and distant organs, especially in tumors greater than 2 to 3 cm.^5^

We present a case of SCAND arising on the scalp of a woman who subsequently developed breast cancer, demonstrating a novel presentation of 2 distinct tumors that can demonstrate challenging and overlapping histopathological features.

## Case report

**Fig 1:**
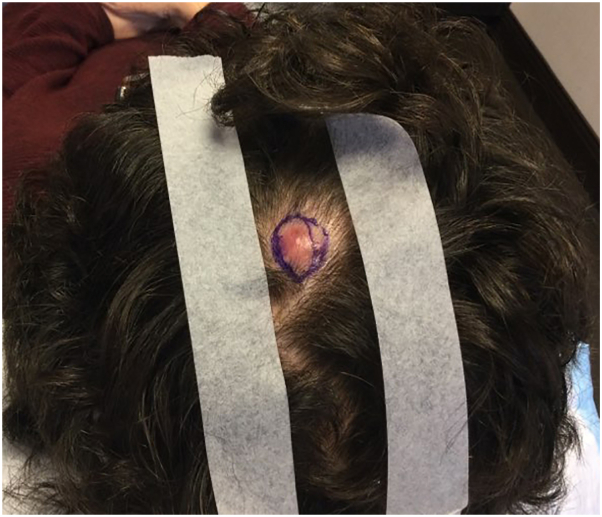
Clinical presentation of SCAND prior to excisional biopsy. Erythematous nodule on the left parietal scalp. *SCAND*, Sweat gland carcinoma with neuroendocrine differentiation.

**Fig 2:**
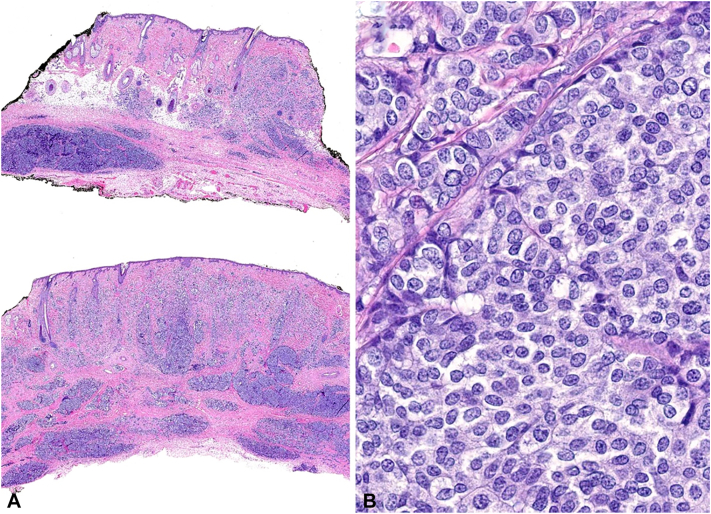
**A,** Histopathology demonstrates a highly infiltrative neoplasm involving the dermis and subcutis. **B,** The tumor cells are round and epithelioid with uniform oval nuclei and powdery chromatin.

**Fig 3:**
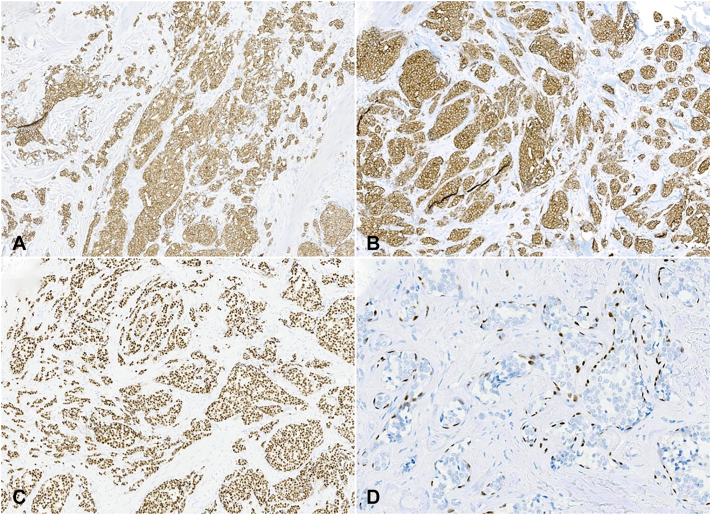
Immunostaining demonstrates diffuse positivity for **(A)** pankeratin, **(B)** synaptophysin, and **(C)** ER. **D,** Focally, p63 highlights a myoepithelial layer around some of the tumor nests, consistent with *in situ* disease. *ER*, Estrogen receptor.

A 73-year-old woman presented with a painless erythematous mass of the left parietal scalp that had been present for 1 year (Fig 1). An excisional biopsy was performed. Histopathology revealed an infiltrative tumor in the dermis, subcutis, and fascia with foci of lymphovascular invasion (Fig 2, *A*). Round cells with neuroendocrine differentiation demonstrated mild nuclear atypia, pale amphophilic cytoplasm, and powdery chromatin (Fig 2, *B*). Immunostaining revealed diffuse positivity for pankeratin, synaptophysin, ER (Fig 3, *A*-*C*), progesterone receptor (PR), GATA binding protein 3 (GATA3), carcinoembryonic antigen (CEA), epithelial membrane antigen (EMA), and androgen receptor. Additional breast and neuroendocrine lineage markers mammaglobin, chromogranin, and CD56 were negative. The tumor cells were also negative for HER2, CK20, SOX10, and p63. Focal tumor nests were surrounded by a layer of myoepithelial cells highlighted by p63 and SOX10 (Fig 3, *D*), supportive of an *in situ* component within sweat glands. Foci of lymphovascular invasion were identified by CD31. While these findings strongly favored a primary diagnosis of SCAND, definitive exclusion of a metastatic carcinoma to the scalp required clinical workup.

Computed tomography demonstrated 2 enhancing left neck level IIb lymph nodes. Computed tomography of the chest, abdomen, and pelvis revealed no additional abnormalities. Mammography was preformed to rule out an occult primary breast cancer and was normal. Exome sequencing revealed no pathogenic variants. Gene expression profiling of the tumor was significant for overexpression of *ESR1, PGR*, *GATA3, TP63,* and *KRT5*. A final diagnosis of SCAND was rendered.

The patient underwent wide local excision and lymph node dissection of the neck, revealing involvement of 2/33 lymph nodes with extranodal extension. Given the estrogen, progesterone, and androgen receptor positivity, she was treated with anastrozole, bicalutamide, and leuprolide. Anastrozole was discontinued after 3 weeks due to a rash. Bicalutamide and leuprolide treatment were continued. She also received adjuvant radiation (66 Gy/33 fractions) to the scalp and bilateral neck nodal basins.

Approximately 8 months after SCAND diagnosis and 3 months after antiandrogen therapy completion, she was diagnosed with stage IA infiltrating ductal carcinoma (ER+/PR−/HER2−/synaptophysin−). This was treated with lumpectomy and radiation, and she was started on exemestane therapy. She remains without SCAND recurrence after 38 months, and without breast cancer for 30 months.

## Discussion

We present a case of metastatic SCAND arising on the scalp of a 73-year-old woman with subsequent breast cancer diagnosis. This case distinguishes the presentation of 2 distinct diseases with overlapping histopathology and immunoreactivity. It further demonstrates sequential and concurrent use of hormonal targeted therapies resulting in remission of both malignancies.

On histopathology, the scalp tumor demonstrated an immunohistochemical profile supportive of an adenocarcinoma (pankeratin+, CEA+) with neuroendocrine differentiation (synaptophysin+) and hormonal receptor positivity (ER+, PR+, androgen receptor positivity). Interestingly, in addition to Merkel cell carcinoma and endocrine mucin-producing sweat gland carcinoma, an important differential diagnosis for this lesion included a breast cancer metastasis to the scalp, as it expressed markers of breast carcinoma including ER, PR, and GATA3.^6^ Although cutaneous metastases from internal malignancies are relatively rare, breast carcinoma is among the most frequent to metastasize to the skin, and the scalp is a frequent site of cutaneous metastasis, likely due to its rich vascular supply.^6^ Thus, additional workup to rule out an internal malignancy was pursued with cross-sectional imaging and mammography and did not reveal an occult primary tumor.

Primary cutaneous sweat gland tumors and metastatic breast cancer demonstrate significant histopathologic overlap (Table I), likely due to the shared similar origin of breast gland and sweat gland tissue from ectodermal appendages.^6^ Notably, GATA3 is expressed in both breast and cutaneous carcinomas, including SCAND. In many cases, histopathology may be insufficient to differentiate these 2 entities, and clinical distinction is required.^6^ Primary cutaneous tumors are more likely to present as a slow-growing solitary nodule, as in this case, whereas cutaneous metastases may grow rapidly with multiple nodules.^6^ The presence of an *in situ* component of malignancy within pre-existing sweat glands strongly favors a primary cutaneous tumor.^5^

**Table I:** Comparison of immunohistochemical staining between the scalp tumor (SCAND) and breast tumor (infiltrating ductal carcinoma)

	Scalp tumor (SCAND)	Breast tumor (Infiltrating ductal carcinoma)
Pankeratin	+	ND
Synaptophysin	+	−
Chromogranin	−	ND
CD56	−	ND
Estrogen receptor	+	+
Progesterone receptor	+	−
HER2	−	−
Androgen receptor	+	ND
GATA3	+	ND
Mammaglobin	−	ND
CEA	+	ND
EMA	+	ND
CK20	−	ND
p63	−∗	ND
SOX10	−∗	ND

*ND*, Not done; *SCAND*, sweat gland carcinoma with neuroendocrine differentiation.

∗ Focal myoepithelial layer only.

Strong expression of hormonal receptors including androgen, estrogen, and progesterone receptors are reported in aggressive sweat gland carcinomas, and management with antiestrogen therapies such as tamoxifen can be effective.^4^^,^^7^^,^^8^ The positive response of our patient to antiandrogen treatment with bicalutamide and leuprolide expands upon these findings, as the use of antiandrogen agents in the treatment of SCAND has not been well described. Notably, this patient developed breast cancer 3 months after treatment completion despite normal mammography at the time of SCAND diagnosis 8 months prior. Although cutaneous metastasis from a radiologically undetectable breast cancer cannot be completely ruled out, it appears unlikely given the different histomorphology of the 2 tumors and the absence of PR and synaptophysin expression in the breast cancer. Presentation of 2 distinct tumors raises the possibility of a genetic predisposition to developing glandular malignancies; however, exome sequencing of the scalp tumor did not reveal any pathogenic variants. Another possibility is that hormonal disruption with antiandrogen therapy accelerated breast cancer development, as the role of androgens in breast cancer growth and development is complex and occasionally conflicting.^9^ Thus, appropriate cancer screening in patients receiving hormonal therapies for cutaneous tumors may be beneficial.

This case highlights the importance of determining hormone receptor expression in SCAND and associated malignancies and provides additional treatment considerations for this rare, aggressive tumor.

## Conflicts of interest

None disclosed.
